# Mental Health in First- and Second-Division Soccer Players: A Cross-Sectional Study

**DOI:** 10.3390/sports12040106

**Published:** 2024-04-11

**Authors:** Lucía Bonet, Ana Benito, Héctor Usó, Marc Peraire, Gonzalo Haro, Isabel Almodóvar-Fernández

**Affiliations:** 1 TXP Research Group, Universidad Cardenal Herrera-CEU, 12006 Castellón, Spainperairemiralles@gmail.com (M.P.); gonzalo.haro@uchceu.es (G.H.); 2 Mental Health Department, Provincial Hospital Consortium of Castellon, 12002 Castellón, Spain; 3 Mental Health Unit Torrente, University General Hospital of Valencia, 46014 Valencia, Spain; 4 Director of Health Department, Villarreal Football Club, 12540 Villareal, Spain; huso@villarrealcf.es; 5 Nursing Department, Jaume I University, 12006 Castellón, Spain; almodova@uji.es; 6 Villarreal Football Club Research Department, 12540 Villareal, Spain

**Keywords:** mental illness, high-performance athletes, football, anxiety, depression

## Abstract

Background: The benefits of sport in mental health have been broadly studied. However, few studies have examined these outcomes in high-performance athletes. We aimed to analyze the state of the mental health of the Villarreal Soccer Club’s first- (FD) and second-division (SD) players and the possible mediating effects of sex and professional category. Methods: This was a cross-sectional study with an initial sample of 108 soccer players (final sample *n* = 54). Data from MINI, HARS, HDRS, BARRAT-11 and SCSRQ questionnaires were analyzed. Results: The mean age was 23.41 years (SD = 4.56) and 61.1% (*n* = 33) were men. A proportion of 24.1% (*n* = 13) stated that they had undergone mental health treatment, 7.4% (*n* = 4) had taken psychotropic drugs, and 2.1% (*n* = 1) had made a suicide attempt. Differences were observed between the FD and SD players in terms of the sensitivity to punishment (t = −2.2; *p* = 0.033), overall impulsivity (t = −3.1; *p* = 0.003), unplanned impulsivity (t = 3.4; *p* = 0.001), and the HDRS (U = −110.5; *p* = 0.004), HARS-Total (U = −104.0; *p* = 0.006) and HARS-Psychological subscale scores (U = −104.0; *p* = 0.001). Differences were also observed between the female and male SD players for the HARS-Somatic subscale (U = 136.5; *p* = 0.028). Conclusion: The low values obtained in the clinical scales, together with the reported psychopathological histories, suggested that the Villareal players showed better mental health than the general population.

## 1. Introduction

Over the last 40 years, interest and concern for health, as well as its relationship with physical activity, has been increasing [1]. According to general recommendations stated by the World Health Organization (WHO), physical activity can have important benefits in terms of the prevention and treatment of cardiovascular or respiratory diseases, among others. This organization has not only highlighted the benefits of sport on physical health but also on mental health for those who engage in physical activities [2]. Mental health is a state of mental wellbeing that enables people to cope with stress, achieve objectives and contribute to community [3]. In this regard, the WHO has highlighted the potential protective effect of exercise against mental pathologies mediated by its capacity to reduce the symptoms of both anxiety and depression [2]. Studies such as the one carried out in Norway by Grasdalsmoen et al. (2022) [4], in China by Zhou et al. (2023) [5], or in the United States by Weber et al. (2023) [6], state that elite university athletes present fewer symptoms of anxiety and depression, have lower suicidal ideation, fewer feelings of loneliness, and greater satisfaction with life in general, when compared to different control groups. However, some of these studies suggest possible differences according to the sex of the athletes, with female athletes presenting more symptoms of both anxiety and depression compared to male athletes [5,6,7]. Moreover, some studies have suggested that the probability of suffering from mental illness may be reduced among athletes involved in team sports compared to those engaging in individual sports [4,8]. An increased sense of belonging and greater accessibility to social support could explain these differences [9,10,11].

Focusing specifically on the mental health of soccer athletes, longitudinal studies have suggested that people who played soccer in their childhood and youth have a lower probability of suffering from depression or suicidal ideation up to 20 years later [12]. It has also been found that former soccer players are half as likely to be admitted for anxiety- and stress-related disorders, alcohol and drug use or affective mood disorders, when compared to the general population [13]. However, other studies have reported a prevalence of the symptoms of depression of between 16.7% and 39% of professional soccer players: an equal or higher prevalence of this symptomatology than that observed in the general population [7,14,15]. There are also studies that suggest that the presence and intensity of the symptoms of depression could be affected by the number of hours dedicated to physical activity, with these symptoms decreasing with the greater the number of hours invested in physical training [4,16]. In contrast, several studies have suggested that both the symptoms of anxiety and depression were more frequent among female team players compared to male team players [6,16].

There is some evidence that the protective effect of physical activity against mental pathologies could be weakened among individuals involved in high-performance sports [17,18]. Indeed, the higher the level of the athletes within their soccer division, the higher their exposure to stressors. Factors such as high levels of pressure to perform, exposure to the media, strong competition, and multiple hours of physical training can all end up impacting the mental health of athletes [19].

In addition, dysfunctional sports environments that provide little support to players may be factors that contribute to the development of maladaptive symptoms or mental disorders of different levels of severity [18]. In this regard, players highlight the relevance of a supportive relationship between players and coaches and between teammates [18,19].

Finally, it must be noted that since the COVID-19 lockdown started, some of these results could have changed. As was found in the general population, many studies described an increased prevalence of depression, anxiety, and stress as a result of the pandemic on athlete populations [20,21]. Despite these rates being lower than expected and some studies pointing out the capacity of the athletes to deal with stress and adverse events [20], it has also been found that athletes with high athletic identity could experience higher levels of anxiety and depression especially when they were unable to participate in their respective sports [22].

Regarding football players, research has reported a perceived decrease in their overall wellbeing which results in a detrimental impact on their general professional performance [23,24]. However, some studies conducted during the lockdown observed that soccer players, compared with individual athletes, had less symptoms of anxiety and depression, suggesting the protective role of team support, as stated before [9].

Taking all the information stated above, it is important to highlight that there is still some relevant controversy regarding the effects of sports on the mental health of high-performance athletes. Nowadays, there are very few studies that have systematically evaluated this issue [25], so further research is needed in order to determine whether the protective effects of physical exercise [3] can be observed in high-performance environments or, on the contrary, if the probability of these athletes suffering severe mental illness is equivalent to or even higher than that of the general population [17]. Thus, the aim of this study was to conduct a descriptive analysis to assess the presence of symptoms associated with mental health disorders among professional players from three different categories in a high-performance soccer team. The specific objectives were to gain insight into the mental wellbeing of these athletes and to examine the possible mediating effect of sex and membership of the first- or second-division categories.

## 2. Methodology

### 2.1. Design

This study employed a cross-sectional and descriptive approach. In this sense, the authors assume a philosophical position derived from the Galilean tradition, with a pragmatic, empirical and quantitative epistemological basis with the objective of producing factual knowledge.

### 2.2. Participants

An initial sample of 108 soccer players from the Villarreal Club de Fútbol (CF) soccer team was selected. The inclusion criteria for the study were (a) age over 18 years, (b) members of a professional federation and active in the first or second division team, (c) adequate oral and written comprehension in Spanish, and (d) having signed the informed consent form to participate in the study. Of the total number of individuals eligible to participate in the study, a final sample of 54 players was included in the subsequent analyses. The remaining participants were excluded because they had not adequately completed all the questionnaires agreed upon. Of these 54 participants, 12 belonged to the men’s first-division team (FDM), 21 to the men’s second-division team (SDM), and another 21 to the women’s second-division team (SDF).

### 2.3. Sociodemographic Data

Regarding the sociodemographic data of the entire cohort of players, we observed that 61.1% (*n* = 33) of them were men and the mean age was 23.41 years (SD = 4.56). Most of the players were single (*n* = 48; 88.9%), lived with their parents (*n* = *n* = 21; 38.9%), did not have children (*n* = *n* = 48; 88.9%) and had completed their studies after completing compulsory secondary education (*n* = *n* = 23; 42.6%).

### 2.4. Comparison of Sociodemographic Data of the Players from the First-Division Team (Male Sex Only) versus the Second-Division Team (Female and Male Sex)

When comparing the data of the players according to whether they belonged to the second (female and male sex) or first (male sex only) division, we observed differences between the groups in terms of marital status, cohabitation status, the number of children they had, and their income level. More information about these results can be found in Table 1.

### 2.5. Comparison of Sociodemographic Data between the Female and Male Second-Division Team Players

Regarding the data for the 42 second-division players (female and male sex), the mean age was 23.24 (SD = 4.54) years and 100% (*n* = 42) of the players were single. Most of them lived with their parents (*n* = 20; 47.6%), did not have children (*n* = 40; 95.2%), and had finished their studies after completing their compulsory secondary education (*n* = 17; 40.5%). When comparing the data for the players in the SDM team to that from the SDF team, we found differences in terms of cohabitation status and socioeconomic level. The data related to this analysis are shown in Table 2.

### 2.6. Procedure

This study was carried out between September 2020 and July 2022. Before beginning data collection, the project was presented to Villarreal CF, and once their permission was obtained, the ethics committee’s authorization was sought. With both authorizations, the recruitment of players began. The project was presented to the rosters of each team, and the coaches later provided us with a list of the players that made up each team. Thus, each player was assigned a code. Football players were asked to sign the informed consent form. Upon delivery of the dossier, the project was once again presented to them, and we addressed every query before the questionnaires were administered. The dossiers coded with the questionnaires that were part of the project were prepared, and a dossier was given to each player. The questionnaires, which were self-administered, were completed by the players themselves at the Villarreal CF facilities. This allowed for any of the present researchers to help resolve any issues that might have arisen while filling out the questionnaires. In the event that they could not complete the questionnaires within the established period of time, the possibility of completing them at home was also offered in exceptional cases. Meanwhile, the research staff administered the questionnaires that were not self-administered. On some occasions, rooms were set up where the players could fill out the questionnaires, and in adjoining rooms, the research staff interviewed them. The research personnel who carried out the evaluation were trained in administering the tests and ensuring concordance.

### 2.7. Instruments

The players were interviewed with the following diagnostic instruments:

- International Neuropsychiatric Interview 5.0 (MINI) [26]: A short, structured diagnostic interview that explores the different axis I psychiatric disorders in the DSM-IV and ICD-10. The MINI is divided into different modules with filter questions corresponding to the diagnostic criteria of each psychiatric pathology. It has adequate reliability and validity, takes less time than other diagnostic interviews [14] and has been used in athletes [27,28]. The reliability in this study was 0.55 (being a cross-sectional study, we have not been able to evaluate test–retest reliability, which would be more appropriate for this instrument).

The following questionnaires were self-completed by the players and supervised by members of the research team:

- Hamilton Anxiety Scale (HARS) [29]: A heteroadministered scale designed to quantify the intensity of the symptoms of anxiety. It consists of 14 items scored on a 0–4 point scale. It makes it possible to obtain a global score that, in turn, can be divided into two scores that refer to anxiety symptoms of a psychic or a somatic nature. The HARS does not have a cut-off point, but the higher the score on the scale, the greater the intensity of the anxiety. It has good psychometric properties, is suitable for clinical and research use [29] and has been used in athletes [30]. The reliability in this study was 0.89.
- Hamilton Depression Scale (HDRS) [31]: A heteroadministered scale designed to quantitatively assess the severity of the symptoms of depression. This was a reduced 17-item version with a response format of 0–4 points. This scale traditionally considers the following cut-off points to establish the severity of the symptoms of depression: 0–7 (absence of depression), 8–13 (slight/minor depression), 14–18 (moderate depression), and >19 (severe depression). It has good concurrent validity, content validity, inter-rater reliability, split-half reliability and alpha reliability [31]. It has been used in athletes [30,32]. The reliability in this study was 0.78.
- Barrat’s Impulsivity Scale (BIS-11) [33]: A self-administered scale for the assessment of impulsivity. The BIS-11 consists of 30 questions grouped into three subscales that assess cognitive, motor and unplanned impulsivity. It has 4 response options that evaluate the frequency of the occurrence of the items. There is no cut-off point, although the validation studies conducted in the Spanish population have established the following medians: cognitive and motor impulsivity: 9.5; unplanned impulsiveness: 14; and total score: 32.5. The BIS-11 has high reliability [34] and has been used in athletes [35,36]. The reliability in this study was 0.78.
- Sensitivity to Punishment and Sensitivity to Reward Questionnaire (SCSRQ) [37]: A self-administered scale with 48 dichotomous items divided into 2 subscales with 24 items each. The first scale assesses sensitivity to punishment and its relationship with the behavioral inhibition system when faced with the possibility of anticipated aversive consequences. The second scale evaluates the sensitivity to reward, related to the behavioral activation system in situations in which the possibility of appetitive or reinforcing stimuli is anticipated. The questionnaire presents satisfactory internal consistency and test–retest reliability [18] and the relationship of sensitivity to punishment and reward with various mental disorders [38,39] and exercise dependence has been studied [40,41]. The reliability in this study was 0.68.

Finally, sociodemographic data about the participants were also collected using the Addictive Disorders Network tool [42].

### 2.8. Data Analysis

To carry out the descriptive analysis of the information obtained in each of the tests, SPSS software (version 23.0; IBM Corp., Armonk, NY, USA) was used. The reliability of the questionnaires was evaluated using Cronbach’s alpha. To analyze the differences between the different groups, chi-squared tests (χ^2^) were applied to compare qualitative variables. When more than 20% of the cells had an expected frequency less than 5, the Monte Carlo test was used as an adjustment. To analyze differences in the quantitative variables, i.e., data from variables that did not meet the assumption of normality or the Student’s *t*-test for normally distributed variables, non-parametric Mann–Whitney U tests were used.

Three types of analyses were performed. First, the joint data from all the players participating in the study were analyzed. Second, to analyze the differences in the mental health of the players according to whether they belonged to the different leagues, data from the players in the FDM team were compared with those from the SDM and SDF combined together. Finally, to analyze the effect of sex on the mental health of the athletes, and with the aim of making the groups as similar as possible, the results of the SDM team were compared with those from the SDF team. Of note, the MINI questionnaire was only administered to the second-division men’s and women’s team players and so these values are only reflected in the comparative table of these two groups.

### 2.9. Ethical Factors

In accordance with Law 14/2007, of July 3, on biomedical research, all the data derived from this study were treated separately from the identity of the group studied, as stated in the Declaration of Helsinki on research with human beings (2013), Regulation (EU) 2016/679 of the European Parliament and of the Council of 27 April 2016, on natural persons with regard to the processing of personal data and their free circulation, and Organic Law 3/2018, of December 5, on Protection of Personal Data and guarantee of digital rights. Additionally, all participants provided their written informed consent, and this study was approved by the Ethics Committee for Biomedical Research at the CEU Cardenal Herrera University (CEI19/134) and is registered at ClinicalTrials.org (NCT04842461) as part of the study “Mental health, addictions and biomarkers in high-performance athletes”.

## 3. Results

### 3.1. Comparison of Psychopathological Data of the Players from the First-Division Team (Male Sex Only) versus the Second-Division Team (Male and Female Sex)

Regarding the clinical data, we observed that 24.1% (*n* = 13) of the players had received mental health treatment, of which 7.4% (*n* = 4) had taken psychotropic drugs and 1.8% (*n* = 1) had made a suicide attempt. The mean total score on the SCSRQ scale was 16.98 (SD = 8.1) and the score on its Sensitivity to Punishment subscale was 8.15 (SD = 4.8), while on the Reward Sensitivity subscale, it was 8.83 (SD = 4.3). Regarding the BARRAT-11 scale scores, the overall mean score was 44.9 (SD = 11.1) and the mean subscale scores were 14.3 (SD = 4.3) for the cognitive impulsivity scale and 16.20 (SD = 5.1) on the unplanned impulsivity scale. The median score on the motor impulsivity scale was 14 (IQR = 7). On the HARS anxiety scale, the overall median was 4.5 (IQR = 7), while the median on the psychological anxiety and somatic anxiety subscales was 3 (IQR = 4) and 1 (IQR = 2), respectively. Finally, the median on the HDRS depression scale was 3 (IQR = 6).

As shown in Table 3, there were significant differences between the first- and second-division players for the following variables: sensitivity to punishment, total impulsiveness, unplanned impulsivity, the overall HARS anxiety scale score, the HARS-psychological subscale score, and the HDRS depression scale score. The values for these variables were higher in the sample of second-division players, except for the differences observed in the impulsiveness variables (Barrat-11), the values of which were higher in the first-division players.

### 3.2. Comparison of Psychopathological Data between the Female and Male Second-Division Team Players

Regarding the clinical data of the 42 second-division players (female and male), 22.2% (*n* = 11) had undergone mental health treatment, of which 9.5% (*n* = 4) had taken psychotropic drugs. None of these players had made any suicide attempts or was in a state of threatening self-harm. The mean total score on the SCSRQ scale was 17.67 (SD = 8.6) and the score on its Sensitivity to Punishment subscale was 8.69 (SD = 5.2), while on the Reward Sensitivity subscale, it was 8.98 (SD = 4.5). Regarding the BARRAT-11 scale scores, the total mean for the scale was 42.64 (SD = 9.8), with mean subscale scores of 13.81 (SD = 3.7) on the cognitive impulsivity scale, 13.81 (SD = 5.8) on the motor impulsivity scale, and 15.05 (SD = 4.3) on the unplanned impulsivity scale. On the HARS anxiety scale, the total median was 5 (IQR = 7), the median on the psychological anxiety subscale was 4 (IQR = 4), and the median on the somatic anxiety subscale was 1 (IQR = 3). The mean total score on the HDRS depression scale was 4.84 (SD = 4.9).

Referring to the MINI scale, we observed that 11 players (26.2%) stated that they had a diagnosis of a mental health disorder. The most frequent being agoraphobia (*n* = 3; 7.3%) and generalized anxiety disorder (*n* = 3; 7.3%), followed by alcohol dependence (*n* = 2; 5%) and bulimia nervosa (*n* = 2; 5%).

When comparing the data for the players in the SDM team to that from the SDF team, significant differences were only observed in the somatic anxiety variable measured by the HARS scale, with higher values in the sample of players from the SDF team compared to the SDM counterparts. No statistically significant differences were found between the rest of the variables analyzed. These data can be consulted in more detail in Table 4.

## 4. Discussion

The results obtained in this cross-sectional study suggest that the Villarreal CF players had better mental health than the general population. This was reflected in the low scores obtained for the HARS anxiety and HDRS depression scales and in the figures for the presence of mental pathology (26.2% vs. 29% in the general population) [43], low use of psychoactive drugs (7.4% vs. 18.1% in the general population) [44], and previous history of self-harm (2.1% vs. 2.7% in the general population) [45]. The results obtained in this study are in line with previous research that has already pointed out the possible protective effect sport might have on the mental health of athletes [4,16]. As stated before, practicing sports and playing on a team could increase access to social support and could promote a sense of belonging, which leads to a better self-stigma and mitigates anxiety and negative mental health symptoms [11].

However, it should be noted that 24.1% of our study sample indicated a previous history of treatment in mental health services. These data are similar to the values registered in the general population, in which 26.2% of Spaniards stated that they are currently receiving psychological (20.8%) or psychiatric (17.6%) treatment [46]. In this sense, performing physical activity within a high-performance context might not imply a reduction in vulnerability to suffering from mental illness because of the exceptional stressors to which players in these teams are exposed [25]. In this regard, some studies highlighted that it is essential to encourage players to develop emotional and cognitive skills to ensure their capacity to deal with the stress of athletic competition [47]. However, although the scores obtained by the players on the SCSRQ for sensitivity to punishment and reward were also within normal limits, the players generally obtained a high score on the BARRAT-11 scale measuring impulsiveness. These measurements were especially high for the unplanned impulsivity subscale. It is important to highlight that high impulsivity scores have been associated with poorer clinical outcomes for multiple mental pathologies, such as affective disorders [48] or substance use disorders [49], among others. In turn, it should be noted that these values were even higher in the sample of first-division players, meaning that this high impulsivity together with the high economic status of these players could increase their predisposition to engage in risky behaviors and their susceptibility to mental health pathologies [48,49]. Nevertheless, the high impulsivity found on this study did not correspond with a high prevalence of mental disorders. In this regard, and contrary to previous studies [48,49], high impulsivity did not seem to be an indicator of poor mental health in our sample. Impulsivity is usually high in athletes, especially in those sports which historically attribute social standing to their players, such as American football, ice hockey, and soccer [50]. Although the data obtained for the variables analyzed were within normal ranges, indicating good mental health in the total sample, it is interesting to note that there were differences according to the division to which the players belonged.

The first division was compared with the second division because previous studies have found differences in mental health between different levels of play, for example, between football players from the first league and the U-21 [16], and between elite and non-elite swimmers [25]. Compared to the first-division players, the players from the second-division teams obtained significantly higher scores on the sensitivity to punishment, total and psychological anxiety, and depression scales. At the same time, the difference between the two categories in terms of treatment history in mental health services also stood out. Only 16.8% of the first-division players had received psychiatric or psychological treatment, compared to 26.2% of the second-division players. These results contradict the approach of previous studies that highlighted an increase in pressure to perform and other stressors with promotion to the first division, which can, ultimately, negatively affect the health of athletes [18,25]. In our sample, the data were always within normative values, but we observed a higher state of psychological wellbeing among players from the first division compared to those belonging to the second division. These data suggest a situation more similar to that defended in the studies by Junge et al. (2016) [16] and Grasdalsmoen et al. (2022) [4], in which a reduction in anxious and depressive symptoms related to the greater number of hours dedicated to training was observed. However, the significant sociodemographic differences between the two groups prevented an adequate comparison of the players from both categories and opened up the possibility that there may have been unanalyzed variables that could have mediated these results. According to data provided by the Spanish Ministry of Health (2017) [44], there is a social gradient both in the frequency of suffering from a mental illness (13.5% in the most disadvantaged social classes compared to 5.9% in favored social classes) and in the risk of suffering from these illnesses (23.6% in disadvantaged social classes compared to 12.4% in the upper social class). In turn, a relationship has been observed between economic instability, concern about said instability, and the greater probability of presenting mental health pathologies, a higher level of consumption of drugs, and the attendance of mental health services (psychology or psychiatry) [46].

Regarding the sex of the athletes, our data contradict some previous studies in which a higher rate of the symptoms of depression and anxiety were found in female rather than male teams [6,16]. According to official studies, anxious and depressive pathologies are found more frequently in the female population than in the male population [43]. However, in our study, we only obtained a significantly higher score in the female team players for the somatic anxiety variable, and we did not observe these differences for the other variables analyzed and the values obtained for both groups were within the normal ranges. Even though the study by Grasdalsmoen et al. (2022) [4] found no differences in terms of mental pathologies according to sex, we were unable to find information that would justify this discrepancy compared to previous publications in which these differences were observed [5,16]. Indeed, factors such as the social support received, history of previous injuries, or the perceived stigma towards mental pathologies, all of which escaped the objectives set out in this current study, could have affected the disparity in our results when compared to previous research [25].

Finally, regarding the effects of the COVID-19 lockdown on mental wellbeing, the reduced rates of depression and anxiety found in our sample suggest that the effects of lockdown could had been buffered by being engaged in a professional team [51]. As stated before, it has been suggested that practicing sports during the lockdown and being part of a professional team could have mitigated the sense of social isolation and may have counteracted the emotional and physical exhaustion produced by the COVID-19 lockdown [51]. Moreover, being used to deal with stress and demanding situations could benefit this group of the population [43].

However, it must be pointed out that since we did not conduct baseline measures to compare these results pre- and post-lockdown, these are hypotheses that should be regarded carefully. As Knowles et al. (2021) [52] stated in their study, we should urge caution with regard to advocating the protective effects of sports during the COVID-19 lockdown, as many other factors such as social support, economic wellbeing, or access to medical services could have explained these positive results.

### Strengths and Limitations

This publication analyzed, for the first time, the state of the mental health of three sections of a professional soccer team belonging to the first and second divisions. In addition, the inclusion of both a women’s and a men’s team in the second division made it possible to compare the results by sex. However, this study had important limitations. Firstly, it is worth noting the large sociodemographic differences observed between the different groups. Specifically, there was a notable difference in terms of the salary received by the first-division players compared to that of the second-division players, which may have meant that these groups were not comparable in terms of the rest of the sociodemographic variables (access to housing or possibility of starting a family, etc.). In addition, these differences suggest that the results obtained for other variables may have been influenced by the characteristics of the samples.

Secondly, a major limitation of this study was the initial reduction in sample size since so many participants had to be eliminated because they had not completed the questionnaires adequately. This study was implemented within the normal operating framework of the team and so the athletes often had to fill out the questionnaires quickly to be able to join their daily training sessions. In the event that they could not complete the questionnaires within the established period of time, the possibility of completing them at home was also offered in exceptional cases. In short, the extra demand that completing the scales during training hours placed on the players, together with the fact that the research team were unable to directly supervise their completion at home, significantly affected the quality of the data obtained. Furthermore, this study was carried out during the COVID-19 pandemic, with the corresponding restrictions, which made sample collection even more difficult. Given the small sample size and, therefore, the small size of the compared groups, the results must be interpreted with caution. In fact, some groups include between 1 and 3 subjects, which prevents correct and adequate analysis, so the reliability of the data is debatable, and no definitive conclusions can be drawn. However, we consider that, given the scarcity of studies on the mental health of elite athletes [25], the results of this study are interesting despite the small sample. In fact, although the study’s response rate (50%) may seem small, response rates in studies in elite athletes can be even lower, at 25% [16]. Furthermore, in the systematic review by Rice et al. (2016) [25], several studies appear with similarly sized or smaller samples than that used in this study. This would highlight the difficulty of recruiting samples from this population.

Another issue to take into account is that some of the instruments used are based on DSM-IV and ICD-10 criteria, while the current classifications are DSM-5 and ICD-11.

Finally, this study did not have a control group with which to compare the results obtained and, in addition, the sample was obtained based on the accessibility the research group had to it. The difficulty of obtaining control groups, as well as obtaining homogeneity in the measurements taken in studies conducted in the field of high-performance sport and especially in football players, has already been pointed out in previous studies [17].

## 5. Conclusions

The results of this study indicate that the Villarreal CF players had better mental health than the general population, regardless of their professional category and sex, which may be related to the health benefits of exercise and sport. However, the greater impulsiveness of the first-division players, increased anxiety and depression in the second-division players, and greater somatic anxiety among the female players were differences that must be considered by the medical departments of soccer clubs.

## Figures and Tables

**Table 1:** Total demographic and comparative data for the first- versus the second-division players.

		TOTAL *n* (%)	First Division (Male Sex Only) *n* (%)	Second Division (Male and Female Sex) *n* (%)	χ^2^ (*p*)
Sex, Men		33 (61.1)	12 (100)	21 (50)	9.8 (0.002) **
Age	Mean (SD)	23.4 (4.6)	24 (4.7)	23.2 (4.5)	14.6 (0.553)
Marital status					23.6 (<0.001) **
	Single	48 (88.9)	6 (50)	42 (100)	
	Married	6 (11.1)	6 (50)	0 (0)	
Living arrangements					16.9 (0.005) **
	Parents	21 (38.9)	1 (8.3)	20 (47.6)	
	Friends	12 (22.2)	1 (8.3)	11 (26.2)	
	As a couple, without children	9 (16.7)	3 (25)	6 (14.3)	
	Other arrangements	12 (22.2)	7 (58.3)	5 (11.9)	
№ of children					7.7 (0.005) **
	None	48 (88.9)	8 (66.7)	40 (95.2)	
	≥1	6 (11.1)	4 (33.3)	2 (4.8)	
Education					5.9 (0.437)
	≤Primary education (6–12 years)	9 (16.7)	4 (33.3)	5 (11.9)	
	Compulsory secondary education (12–16 years)	23 (42.6)	6 (50)	16 (38.1)	
	Undergraduate degree (unfinished)	8 (14.8)	1 (8.3)	7 (16.7)	
	Undergraduate degree (completed)	7 (13)	1 (8.3)	6 (14.3)	
	Others	7 (13)	0 (0)	7 (13.0)	
Employment status	Temporary contract	54 (100)	12 (100)	42 (100)	-
Income					54.0 (<0.001) **
	<EUR 450	10 (18.5)	0 (0)	10 (23.8)	
	EUR 450–900	13 (24.1)	0 (0)	13 (31.0)	
	EUR 900–1500	14 (25.9)	0 (0)	14 (33.3)	
	EUR 1500–2700	5 (9.3)	0 (0)	5 (11.9)	
	>EUR 3600	12 (22.2)	12 (100)	0 (0)	
Total		54	12	42	

** *p* < 0.01.

**Table 2:** The demographics of the men’s and women’s second-division teams.

		TOTAL *n* (%)	SDF ^1^ *n* (%)	SDM ^2^ *n* (%)	χ^2^ (*p*)
Age	Mean (SD)	23.2 (4.5)	23 (5.2)	23.5 (3.8)	19.3 (0.252)
Marital status	Single	42 (100)	21 (100)	21 (100)	-
Living arrangements					11.9 (0.036) *
	Parents	20 (47.6)	7 (33.3)	13 (61.9)	
	Friends	11 (26.2)	9 (42.9)	2 (9.5)	
	As a couple, without children	6 (14.3)	4 (19.0)	2 (9.5)	
	Other arrangements	5 (11.9)	1 (9.5)	4 (19.0)	
№ of children					2.1 (0.147)
	None	40 (95.2)	21 (100)	19 (90.5)	
	≥1	2 (4.8)	0 (0)	2 (9.5)	
Education					4.9 (0.562)
	≤Primary education (6–12 years)	5 (11.9)	1 (4.8)	4 (19.0)	
	Compulsory secondary education (12–16 years)	17 (40.5)	7 (33.3)	9 (42.9)	
	Undergraduate degree (unfinished)	7 (16.7)	4 (19.0)	3 (14.3)	
	Undergraduate degree (completed)	6 (14.3)	4 (19.0)	3 (14.3)	
	Others	7 (16.7)	5 (23.8)	2 (9.5)	
Employment status	Temporary contract	42 (100)	21 (100)	21 (100)	
Income					29.2 (<0.001) **
	<EUR 450	10 (23.8)	9 (42.9)	1 (4.8)	
	EUR 450–900	13 (31.0)	10 (47.6)	3 (14.3)	
	EUR 900–1500	14 (33.3)	0 (0)	14 (66.7)	
	EUR 1500–2700	5 (11.9)	2 (9.5)	3 (14.3)	
Total		42	21	21	

* *p* < 0.05, ** *p* < 0.01. ^1^ SDF: second-division female. ^2^ SDM: second-division male.

**Table 3:** Overall clinical data and comparative data for the first versus the second division.

		TOTAL	First Division (Male Sex Only)	Second Division (Male and Female Sex)	χ^2^ (*p*)
		***n*** **(%)**	***n*** **(%)**	***n*** **(%)**	**χ^2^ (*p*)**
Mental health treatment		13 (24.1)	2 (16.8)	11 (26.2)	0.5 (0.496)
Psychotropic drugs taken		4 (7.4)	1 (8.3)	0 (0)	4.6 (0.098)
Self-harm threatened		1 (1.8)	1 (8.3)	0 (0)	3.6 (0.059)
Suicide attempt		1 (1.8)	1 (8.3)	0 (0)	3.6 (0.060)
		**Mean (*SD*)** **Median (IQR)**	**Mean (*SD*)** **Median (IQR)**	**Mean (*SD*)** **Median (IQR)**	**Student’s *t*-test (*p*) ^1^** **Mann–Whitney U test (*p*) ^2^**
SCSRQ	SCSRQ-Total	16.9 (8.1)	14.0 (5.1)	17.44 (8.8)	−1.2 (0.244)
	Sensitivity to Punishment	8.2 (4.8)	6.1 (2.7)	8.6 (5.3)	−2.2 (0.033) *
	Sensitivity to Reward	8.8 (4.3)	7.9 (3.8)	8.9 (4.3)	−0.5 (0.655)
BARRAT-11	Impulsivity-Total	44.9 (11.1)	53.6 (12.4)	42.1 (9.8)	3.1 (0.003) **
	Impulsivity-Cognitive	14.3 (4.3)	16.9 (5.4)	13.4 (3.5)	1.6 (0.109)
	Impulsivity-Motor	14 (7)	15 (10)	13 (7)	178.0 (0.123)
	Impulsivity-Unplanned	16.2 (5.1)	20.68 (5.9)	15.03 (4.2)	3.4 (0.001) **
HARS	HARS-Total	4.5 (7)	1 (4)	6 (7)	104.0 (0.006) **
	HARS-Psychological	3 (4)	1 (3)	4 (4)	104.0 (0.006) **
	HARS-Somatic	1 (2)	0 (1)	1 (3)	157.5 (0.091)
HDRS	HDRS-Total	3 (6)	1 (2)	4 (5)	110.5 (0.004) **
Total		54	12	42	

* *p* < 0.05, ** *p* < 0.01. ^1^ Mann–Whitney U test: represented by the median and interquartile range (IQR): Md (Q1–Q3). ^2^ Student’s *t*-test: represented by the mean and standard deviation: X (SD).

**Table 4:** Clinical data from the male and female second-division teams.

		TOTAL	SDF ^1^	SDM ^2^	
		*n* (%)	*n* (%)	*n* (%)	χ^2^ (*p*)
Mental health treatment		11 (26.2)	7 (33.3)	4 (19.0)	1.1 (0.292)
Psychotropic drugs taken		4 (9.5)	3 (14.3)	1 (4.8)	1.1 (0.293)
Threatening self-harm		0 (0)	0 (0)	0 (0)	-
Suicide attempt		0 (0)	0 (0)	0 (0)	-
		**Mean (*SD*)** **Median (IQR)**	**Mean (*SD*)** **Median (IQR)**	**Mean (*SD*)** **Median (IQR)**	**Student’s *t*-test (*p*) ^3^** **Mann–Whitney U test (*p*) ^4^**
SCSRQ	SCSRQ-Total	17.7 (8.6)	12.3 (7.6)	18.8 (10.1)	−1.1 (0.303)
	Sensitivity to Punishment	8.7 (5.2)	8.3 (5.2)	8.8 (5.6)	−0.4 (0.663)
	Sensitivity to Reward	8.9 (4.5)	7.9 (3.7)	9.9 (5.4)	−1.5 (0.142)
BARRAT-11	Impulsivity-Total	42.6 (9.8)	41.9 (10.2)	42.2 (9.6)	−0.5 (0.654)
	Impulsivity-Cognitive	13.8 (3.7)	13.6 (3.5)	13.2 (3.4)	−0.4 (0.681)
	Impulsivity-Motor	13.8 (5.8)	14.0 (6.5)	13.2 (5.4)	0.2 (0.834)
	Impulsivity-Unplanned	15.0 (4.3)	14.4 (11.9)	15.7 (4.9)	−0.9 (0.362)
HARS	HARS-Total	5 (7)	6 (8)	3.5 (6)	144.5 (0.86)
	HARS-Psychological	4 (4)	4 (4)	3 (5)	166.0 (0.248)
	HARS-Somatic	1 (3)	2 (5)	0 (2)	136.5 (0.028) *
HDRS	HDRS-Total	4.8 (4.9)	8.8 (9.1)	5.1 (4.3)	0.7 (0.499)
MINI	Diagnosis	11 (26.2)	4 (19.0)	7 (33.3)	
	Depression/Dysthymia	1 (2.4)	0 (0)	1 (4.8)	0.9 (0.323)
	Manic–hypomanic episode	1 (2.4)	0 (0)	1 (4.8)	0.9 (0.323)
	Panic disorder	1 (2.4)	0 (0)	1 (4.8)	0.9 (0.323)
	Agoraphobia	3 (7.3)	1 (5.0)	2 (9.5)	0.3 (0.578)
	Post-traumatic stress disorder	1 (2.4)	0 (0)	1 (4.8)	0.9 (0.323)
	Alcohol dependency	2 (5.0)	2 (5.0)	2 (9.5)	2.0 (0.157)
	Bulimia nervosa	2 (5.0)	2 (10.0)	0 (0)	2,1 (0.147)
	Generalized anxiety disorder	3 (7.3)	0 (0)	3 (15.0)	3.2 (0.072)
Total		42	21	21	

* *p* < 0.05. ^1^ SDF: second-division female. ^2^ SDM: second-division male. ^3^ Student’s *t*-test: represented by the mean and standard deviation: X (SD). ^4^ Mann–Whitney U test: represented by the median and interquartile range (IQR): Md (Q1–Q3).

## Data Availability

Data are contained within the article.
